# Sex Influence on Autophagy Markers and miRNAs in Basal and Angiotensin II-Treated Human Umbilical Vein Endothelial Cells

**DOI:** 10.3390/ijms241914929

**Published:** 2023-10-05

**Authors:** Flavia Franconi, Giampiero Capobianco, Giuseppe Diana, Valeria Lodde, Alberto De Donno, Maria Laura Idda, Andrea Montella, Ilaria Campesi

**Affiliations:** 1 Laboratory of Gender Medicine, National Institute of Biostructures and Biosystems, 07100 Sassari, Italy; franconi.flavia@gmail.com; 2 Department of Medicine, Surgery and Pharmacy, Gynecologic and Obstetric Clinic, University of Sassari, 07100 Sassari, Italy; capobia@uniss.it (G.C.); albertoloris@gmail.com (A.D.D.); 3 Department of Biomedical Sciences, University of Sassari, 07100 Sassari, Italyvlodde@uniss.it (V.L.); 4 Institute of Genetics and Biomedical Research, National Research Council, 07100 Sassari, Italy; m.laurai@yahoo.it

**Keywords:** HUVEC, sex differences, angiotensin II, autophagy, mitophagy, Beclin-1, p62, LC3, LAMP-1, parkin, miRNA

## Abstract

Cardiovascular diseases (CVD) display many sex and gender differences, and endothelial dysfunction, angiotensin II (Ang II), and autophagy represent key factors in the autophagic process Therefore, we studied whether Ang II modulates the mentioned processes in a sex-specific way in HUVECs obtained from healthy male and female newborns. In basal HUVECs, the Parkin gene and protein were higher in FHUVECs than in MHUVECs, while the Beclin-1 protein was more expressed in MHUVECs, and no other significant differences were detected. Ang II significantly increases LAMP-1 and p62 protein expression and decreases the expression of Parkin protein in comparison to basal in MHUVECs. In FHUVECs, Ang II significantly increases the expression of Beclin-1 gene and protein, and *Parkin* gene. The LC3 II/I ratio and LAMP-1 protein were significantly higher in MHUVECs than in FHUVECs, while Parkin protein was significantly more expressed in Ang II-treated FHUVECs than in male cells. Ang II affects the single miRNA levels: miR-126-3p and miR-133a-3p are downregulated and upregulated in MHUVECs and FHUVECs, respectively. MiR-223 is downregulated in MHUVEC and FHUVECs. Finally, miR-29b-3p and miR-133b are not affected by Ang II. Ang II effects and the relationship between miRNAs and organelles-specific autophagy is sex-dependent in HUVECs. This could lead to a better understanding of the mechanisms underlying sex differences in endothelial dysfunction, providing useful indications for innovative biomarkers and personalized therapeutic approaches.

## 1. Introduction

Cardiovascular diseases (CVD) are the primary cause of death worldwide [1]. Importantly, there are numerous sex and gender differences in presentation, diagnosis, natural course, treatments, and outcomes of CVD such as hypertension, ischemic heart disease, heart failure, vascular complications of diabetes mellitus, ictus, etc. [2,3,4]. Notably, endothelial dysfunction is a hallmark of CVD, and is also a predictor of CVD outcomes [5]. It has been reported that microvascular dysfunction may contribute to ventricular remodelling and leads to an increased likelihood of heart failure hospitalizations [6,7]. Furthermore, endothelial dysfunction can promote atherosclerosis, increasing morbidity and mortality for severe cardiovascular events [8]. This is especially evident in women [9], and some of the sex differences in CVD can be attributed to differences in endothelial dysfunction [10].

The endothelium plays a key role in vascular and immune functions, being involved in blood pressure regulation, angiogenesis, thrombotic state, atherosclerosis and inflammation, etc. [11,12]. Endothelial functions are governed by numerous factors such as lifestyle, behavioral factors, gut microbiota, sympathetic and immune activation, sexual and vasoactive hormones such as the renin–angiotensin–aldosterone system (RAAS) [13,14,15,16]. Preclinical and clinical studies support the hypothesis that RAAS mainly contributes to the pathogenesis of some CVDs. For example, angiotensin II (Ang II) accelerates hypertension [17] and promotes atherosclerosis [18] and endothelial dysfunction [19]. Moreover, it has been reported that Ang II and other components of RAAS can operate in a sex-dependent manner [2,20,21,22].

A process that contributes to sex differences in the physiology and pathophysiology of CVD is autophagy [23,24,25,26,27]. Physiologically, this cannibalistic process degrades and recycles cellular components providing essential nutrients for cell survival [28], while excessive or defective autophagic activity is not compatible with cellular and organismal homeostasis, and aberrant autophagy is generally associated with a wide range of pathologies [23,24]. Autophagy involves three stages: induction of autophagy, nucleation of the autophagosome, and elongation of the autophagosome [28], which are characterized by specific markers. Briefly, Beclin-1 is involved in the formation of the pre-autophagosome, LC3I and its delipidated form LC3II are involved in the autophagosome formation [28], and the lysosomal membrane protein LAMP-1 is involved in autophagosomes fusion with endosomes/lysosomes [28].

Selective forms of autophagy that degrade specific organelles as damaged mitochondria (mitophagy) are also described [28]. Mitophagy mainly occurs through ubiquitin-mediated mitophagy, which is linked to Parkin, which acts as a downstream protein of PINK1 [29]. In the early 1960s, the first sex differences in mitochondria were described, and a multitude of differences have been found, which have been reviewed in detail [30]. In some organs and tissues, mitophagy is influenced by sex, but data are not available in HUVECs, although aberrant mitophagy has been related to numerous CVDs including atherosclerosis [29,31,32]. Progress has been made in mechanisms governing selective autophagy receptors such as p62. The multifunctional protein adaptor p62 participates in mitophagy and cargo autophagy and has many other activities [29]. For example, disturbance of mitophagy is accomplished by an accumulation of p62, which is responsible for bringing polyubiquitinated proteins to the autophagosome [32].

It has recently been discovered that the different steps of the selective and non-selective autophagic process can be finely regulated by micro-RNAs (miRNAs) [33] whose levels, in some cases, may be regulated by sexual hormones [34]. Some miRNAs can inhibit the initial formation stage of autophagosomes by inhibiting the expression of *Beclin-1*, while others inhibit the elongation stage by working directly on *LC3* [33]. For example, the role of miR-133a in the regulation of mitophagy was demonstrated in mice [35]. Therefore, it is emerging that some miRNAs are new players in the regulation of autophagy, and alterations of autophagy-related miRNAs are implicated in several pathological conditions like cancer, neurodegeneration, CVD, and infections [36]. Interestingly, autophagy may itself modulate miRNA levels, as they are selectively sequestered and degraded by the lysosomal pathway [33]. In addition, miRNAs act on RAAS [37,38], and their levels are modified during hypertension [39,40]. The exact role of Ang II in endothelial autophagy has not been fully clarified, although autophagy may play a key role in CVD, including endothelial dysfunction [32,41]; even less is known whether these activities of Ang II are influenced by sex.

HUVECs are a useful model to study endothelial function and properties [42]. A certain number of studies have shown sex differences regarding gene expression, protein expression, cell viability after serum starvation, tube formation capacity, constitutive and stimulated autophagy, intracellular ATP and metabolite levels, and oxidative stress land angiogenesis on HUVECs [25,41,43,44,45,46,47].

It has become crucial to study sex’s influence on autophagy in primary HUVECs looking at genes, miRNAs, and proteins either in basal or in Ang II-treated cells. This knowledge will improve our therapeutic approaches in CVD because until now, in the majority of cases, men, male animals, and cells of unknown sex have been used, generating scarce knowledge of female needs in health and diseases.

## 2. Results

### 2.1. Characteristics of Donors

The mothers of female and male neonates did not differ significantly in age, body weight at the beginning and the end of the pregnancy, and body mass index. Neonates of both sexes did not diverge significantly in gestational age and body weight at birth (Table 1).

### 2.2. LDH Release and MDA Levels

In basal male and female cells, LDL release was similar, as were MDA levels. In male and female HUVECs (MHUVECs and FHUVECs, respectively), the different doses of Ang II did not significantly change the release of LDH, and MDA levels were not changed by 0.5 µM (Figure S1).

### 2.3. Viability

**Figure 1:**
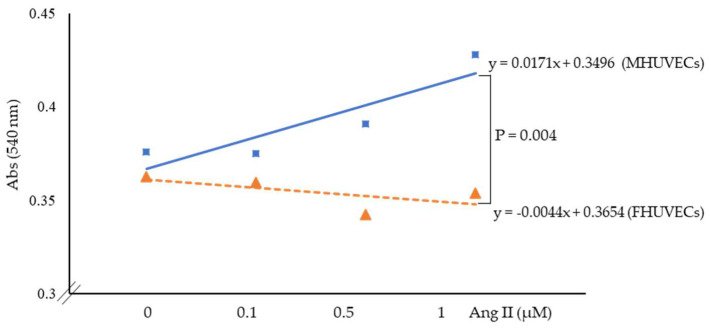
Linear regression analysis of the effect of angiotensin II on the viability of MHUVECs (light blue) and FHUVECs (orange). Data are reported as mean ± SD of at least 5 subjects per sex.

In basal cells, viability was not statistically significantly different in the two sexes (Figure 1). The dose–response curve of Ang II showed a clear sex effect (Figure 1). In particular, the treatment with Ang II of FHUVECs had no significant effect on viability, although there was a tendency to decrease when compared to basal cells. In MHUVECs, the treatment with Ang II dose-dependently increased the viability (Figure 1). Linear regression analysis showed that slopes significantly diverged between MHUVECs and FHUVECs (Figure 1).

### 2.4. Effect of Ang II on HUVECs Migration

**Figure 2:**
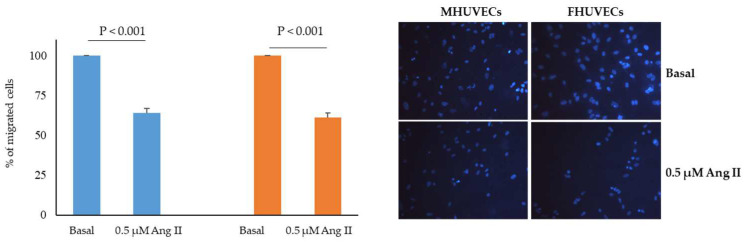
Effect of Ang II on the migration of MHUVECs (light blue) and FHUVECs (orange). Data are reported as mean ± SD of at least 5 subjects per sex. Representative photographs were taken at ×20 magnification.

In basal conditions, male and female HUVECs did not present any significant difference in the number of migrated cells (4983 ± 472 cells in FHUVECs and 6630 ± 760 in FHUVEC, *n* = 5 for each sex; *p* = 0.79). In both male and female HUVECs, treatment with Ang II significantly reduced migration to a similarl degree (by about 35% and 40% in MHUVECs and FHUVECs, respectively) (Figure 2).

### 2.5. Effect of Ang II on Macroautophagy and Mitophagy

**Figure 3:**
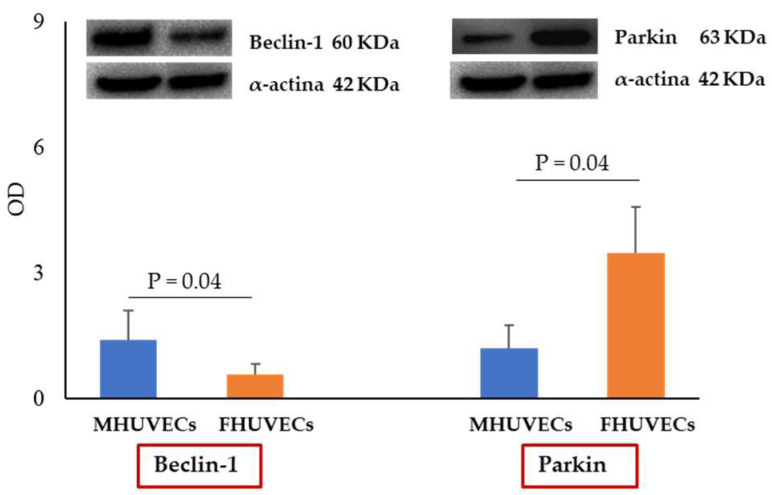
Densitometric analysis and representative Western blotting images of Beclin-1 and Parkin protein expression in basal conditions in MHUVECs (light blue) and FHUVECs (orange). Data are reported as mean ± SD of at least 5 subjects per sex.

Macroautophagy and mitophagy were evaluated through the analysis of gene and protein expression of the main molecules involved in these processes. Under basal conditions, no significant differences between MHUVECs and FHUVECs were observed in the gene expression of *Beclin-1*, *LC3I*, *LAMP-1*, *p62*, or *Parkin*, while the protein expression of Beclin-1 was significantly higher in males (2.4 fold increase) and that of Parkin was higher in females (2.9 fold increase); the other proteins did not diverge (Figure 3).

**Figure 4:**
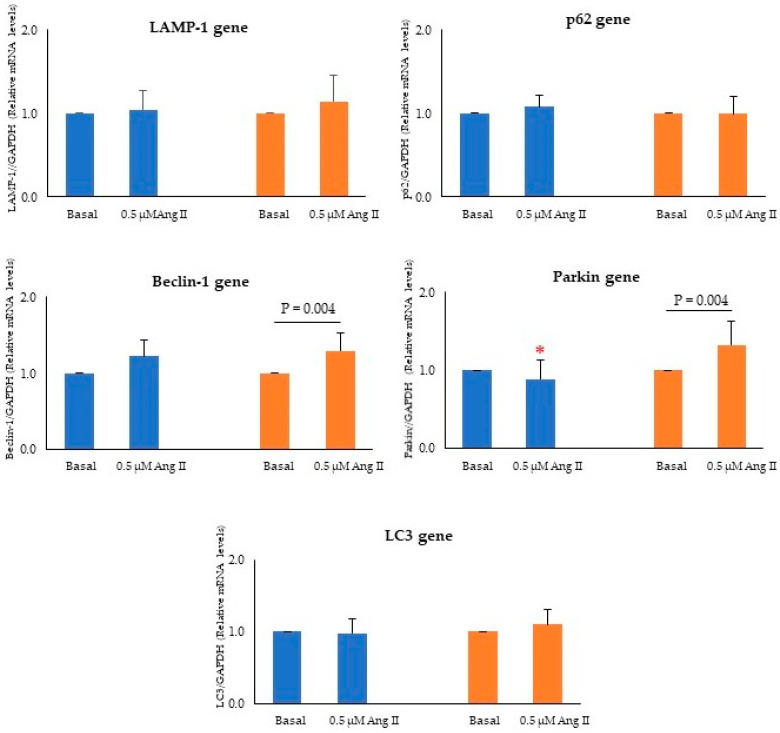
Relative gene expression of *LAMP-1*, *p62*, *Beclin-1*, *Parkin*, and *LC3* in MHUVECs (light blue) and FHUVECs (orange) before and after Ang II treatment. Data are reported as mean ± SD of at least 5 subjects per sex. * *p* = 0.03 versus Ang II-treated FHUVECs.

Ang II treatment did not affect gene expression in MHUVECs (Figure 4), while it resulted in significant increases in *Beclin-1* and *Parkin* levels in FHUVECs compared to baseline. Moreover, FHUVECs showed significantly higher levels of *Parkin* gene than MHUVECs (1.5 fold increase) (Figure 4).

**Figure 5:**
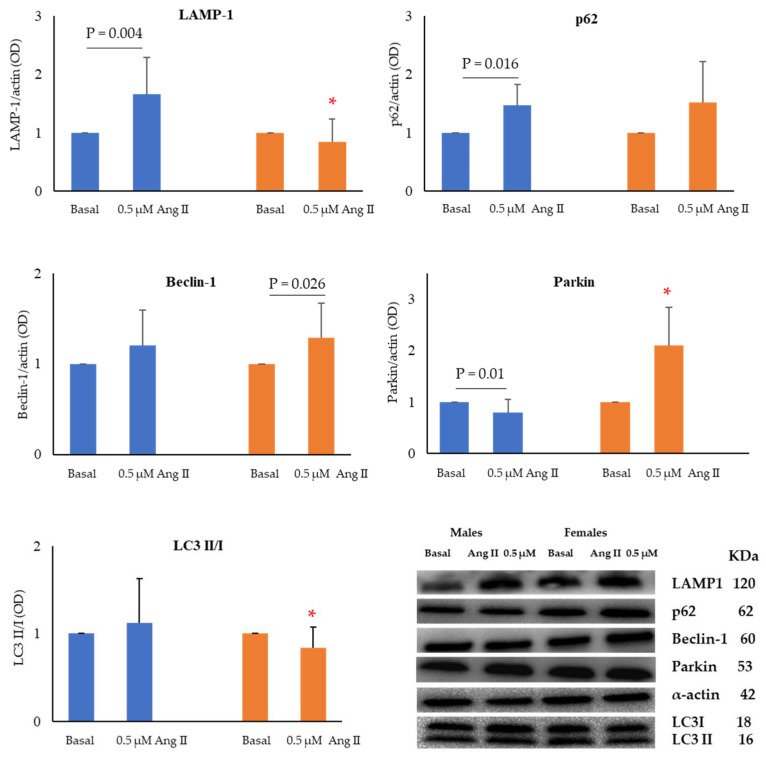
Densitometric analysis and representative Western blotting images of LAMP-1, p62, Beclin-1, and Parkin proteins, and LC3 II/I ratio in MHUVECs (light blue) and FHUVECs (orange) before and after Ang II treatment. Data are reported as mean ± SD, calculated as an increase over the control of at least 5 subjects per sex. * *p* = 0.03 versus Ang II-treated MHUVECs.

In addition, in male cells, the treatment with Ang II caused an increase in p62 and LAMP-1 protein expression and a decrease in Parkin levels compared to basal conditions (Figure 5). A significant increase in the expression of Beclin-1 protein was instead found in Ang II-treated FHUVECs than in untreated cells (Figure 5).

Finally, the LC3 II/I ratio and LAMP-1 protein were significantly higher in MHUVECs than in FHUVECs (1.3 and 1.98 fold increase, respectively), while the Parkin protein was significantly more expressed in Ang II-treated FHUVECs than in male cells (2.6 fold increase) (Figure 5).

### 2.6. Effect of Ang II on miRNAs

The following miRNAs were measured: miR-29b-3p, miR-126-3p, miR-133a-3p, miR-133b, and miR-223-3p. Under basal conditions, levels of miRNAs were similar in both MHUVECs and FHUVECs.

**Figure 6:**
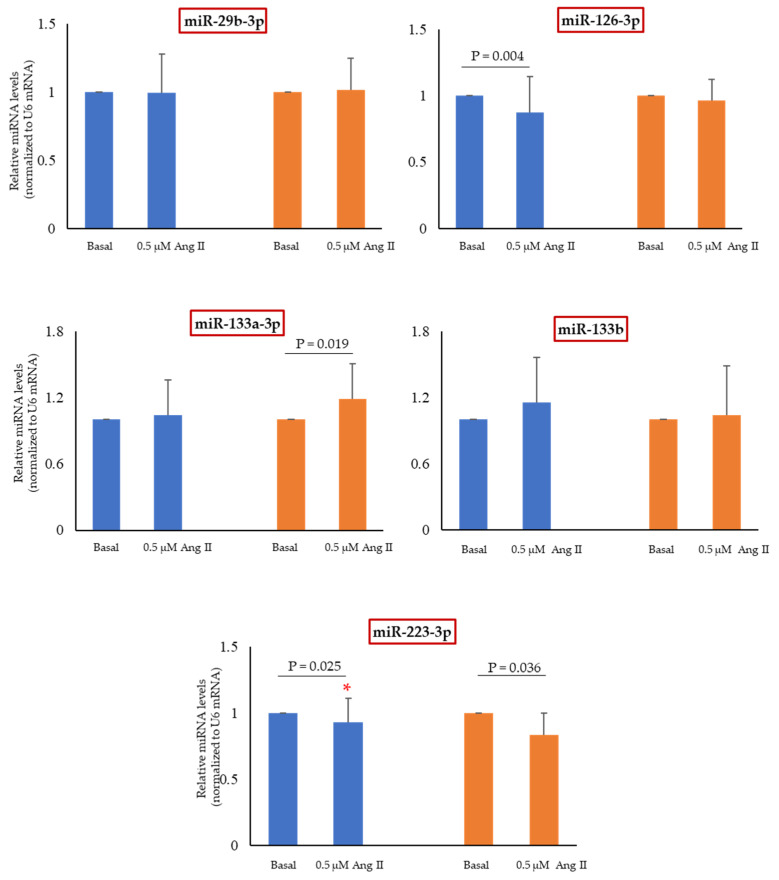
Relative expression of miR-29b-3p, miR-126-3p, miR-133a-3p, miR-133b, and miR-223-3p in MHUVECs (light blue) and FHUVECs (orange) before and after Ang II pre-treatment. Data are reported as mean ± SD of at least 5 subjects per sex. * *p* = 0.03 versus II-treated female HUVECs.

Ang II affected the single miRNA level in a sex-dependent way (Figure 6): miR-126-3p was downregulated only in MHUVECs, while miR-133a-3p was upregulated only in FHUVECs. MiR-223-3p was downregulated by Ang II both in male and female cells, being the reduction more pronounced in FHUVECs. Finally, miR-29b-3p and miR-133b were not affected by Ang II.

## 3. Discussion

Endothelial cells are involved in numerous physiological and pathological processes [5,10,11,12,48] and Ang II is a crucial player in the physiology and physiopathology of the endothelium [13,17,18,19].

Notably, sex influences both activities of endothelial cells and Ang II [20,21,22,25,46]. The results of the present study indicate that some sex differences are present in basal cells, but the major novelties are linked to exposure to Ang II. In fact, the polypeptide induced qualitative and quantitative sex differences. In particular, for the first time, it has been demonstrated that Ang II induces cell autophagy in HUVECs in a sex-dependent manner.

In basal HUVECs, the only sex differences measured regard Parkin gene and protein, which are more expressed in FHUVECs than MHUVECs, and the expression of Beclin-1 protein, which is more elevated in MHUVECs than in FHUVECs. The higher expression of Beclin-1 in basal male cells confirms the previous data [25]; however, previously, it was found that the LC3II/LC3I ratio was higher in MHUVECs than in FHUVECs [25]. Instead, here, the LC3II/LC3I ratio is similar in both FHUVECs and MHUVECs. The contradictory results could depend on the demographic characteristics of mothers. Here, they are older and weigh less than in the previous paper, and it is known that autophagic activity fits with metabolic profile and age [49,50].

Ang II elevates sex differences from two to five when compared to basal cells. Among the parameters that become different with sex, there is cell viability: in FHUVECs, there is a small but not significant decrease in cell viability, whereas in male cells, it is significantly increased. This is in line with previous HUVEC cell lines of unknown sex [51,52], but in the majority of cases, it was shown that Ang II induced viability loss in HUVEC cell lines of unknown sex (an exhaustive list of references about this point is reported in Table S3 [53,54,55,56,57,58,59,60,61,62,63,64,65,66]). The discrepancy could be attributed to the use of HUVEC cell lines versus the primary cultures we used, and to different methods of culture and viability determination.

Interestingly, both in basal and Ang II-stimulated FHUVECs there is an enhancement of autophagy occurring in a major elimination of the dysfunctional mitochondria. Several sex differences in female and male mitochondria are reported during postnatal life [67,68,69]; here, it is evident that mitochondrial sex differences start in prenatal life either in basal or Ang II-treated cells. In particular, Ang II promotes the expression of both the Beclin-1 and Parkin genes and proteins in FHUVECs, whereas the p62 and LAMP-1 proteins are similar in basal and Ang II-treated female cells. These findings suggest that Ang II in FHUVECs is a regulator of autophagy being the effect focused on the initiation of the process and on mitochondrial autophagy. This is in line with the fact that Ang II is a facilitator of the translocation of PARK2 into mitochondria, where it co-localizes into Parkin-positive mitochondria [70], suggesting a potential role for Beclin-1 in linking mitochondria to autophagosomes [71]. Similar results were obtained in rat hearts [72] and in immortalized mouse podocytes of unknown sex [73].

In MHUVECs, Ang II increases the expression of LAMP-1 and p62 protein and decreases the expression of Parkin protein. The multifactorial protein p62 participates in the autophagic process as a lysophagy adapter [74], essential to the selective autophagy of protein aggregates and Parkin-independent mitochondria autophagy [75]. In addition, the increase in p62 levels inside cells is associated with a defect in the final steps of autophagy [28]. p62 binds to LC3 and co-localizes with LC3 and LAMP-1, so therefore may also be used to monitor the autophagic flux [28]. The LAMP-1 protein is required for autophagosomes’ fusion with endosomes/lysosomes [28]. Globally, the influence of “male sex” is more evident when the final steps of the process are considered.

Ang II affects the levels of some miRNAs such as miR-126-3p (one of the most abundant miRNAs in endothelial cells involved in vascular remodeling [76,77]), whose levels are reduced only in MHUVECs, while miR-133a-3p (a cardiomyocytes-derived miRNAs and one of miRNA linked to angiogenesis [78]) is upregulated only in FHUVECs. Plasma miR-126 increases also in hypertensive patients with albuminuria, and miR-126-3p appears to be a good predictor of endothelial dysfunction, which is also associated with a shorter time free of both cardiovascular events, including coronary artery disease and stroke [79].

Previously, it was shown that expression of miR-133a-3p was reduced in a dose-dependent manner by Ang II in a cell line of HUVECs of unknown sex inducing apoptosis [80]. In line with previous data [81], miR-223-3p is expressed in freshly isolated endothelial cells. It is involved in invasiveness, immunity, and metabolism-related disorders such as diabetes mellitus [82,83]. It is also considered an independent, non-invasive biomarker of atherosclerosis and/or type 2 diabetic patients [82]. Its levels are reduced by Ang II both in male and female cells, indicating that it is a target of Ang II. Finally, miR-29b-3p (anti-fibrotic miRNA deriving from cardiac and fibroblast cells, and a key regulator of angiogenesis [84,85]) and miR-133b (a cardiomyocyte-derived miRNA and a factor involved in atherosclerosis [86]) were unchanged in both MHUVECs and FHUVECs.

The individual miRNAs may affect different steps of the autophagic process. The increase in levels of miR-133a-3p observed in FHUVECs is associated with an increase in the Parkin and Beclin-1 proteins, whereas the decrease in miR-126-3p in MHUVECs is associated with an increase in p62 and LAMP-1. Also, Yang et al. [33] described that some miRNAs (miR-376, miR-17, and miR-216a, for example) can inhibit the initial formation stage of autophagosomes, while others (miR-204, for example) inhibit the elongation stage.

In light of what has been reported, we can state that the relationship between miRNAs and organelle-specific autophagy after treatment with Ang II is influenced by sex. Thus, the effect of Ang II depends on a single miRNA and the sex of cells, indicating the strategic relevance of sex. A deeper knowledge of the influence of sex on the interaction between autophagy, miRNAs, and RAAS will give useful insights into the mechanisms of endothelial dysfunction and underline that further investigations will provide insights into innovative pharmacological approaches and biomarkers to target RAAS-mediated CVD and obtain a personalized treatment.

## 4. Materials and Methods

### 4.1. Donors

Umbilical cords were obtained from healthy Caucasian human male and female neonates vaginally delivered at term (37–42 weeks) at the Obstetrics and Gynaecology Clinic, University of Sassari. The mothers were healthy, non-obese, non-smoking, and drug-free, except for folic acid and iron supplementation, and had uncomplicated pregnancy. HUVECs were obtained only from umbilical cords of normal-weight neonates according to Bertino et al. [87] (2430–4050 and 2550–4190 g for males and females, respectively, which represented the 10th and 90th centiles in Ines charts), and they were isolated within 24 h of spontaneous delivery. Informed consent was obtained from the mothers of all subjects donating umbilical cords following the Declaration of Helsinki.

### 4.2. Experimental Model 

The perturbation induced by Ang II in HUVECs is considered a good model for studying endothelial functions [42]. Male and female basal groups (HUVECs cultivated without treatments) and male and female Ang II-treated HUVECs at concentrations indicated in tables and figures for 24 h were used. Some pilot experiments were performed to highlight whether Ang II had the same cytotoxic effects in male and female cells measuring lactate dehydrogenase (LDH) and lipid peroxidation as malondialdehyde (MDA) levels.

In addition, it was investigated whether the housekeeping gene glyceraldehyde-3-phosphate dehydrogenase (*GAPDH*) has similar values in male and female cells. The values measured value were 18.27 ± 0.20 mean Ct for female cells, and 18.07 ± 0.30 for males (*n* = 5 for each sex; *p* = 0.21). Furthermore, pilot experiments showed that the housekeeping gene *U6*, which is normally used to normalize miRNAs data, did not differ between male and female cells (14.56 ± 0.84 mean Ct for female cells, and 14.68 ± 0.40 for male cells, *n* = 5 for each sex; *p* = 0.76).

### 4.3. Cell Isolation and Characterization

Umbilical cords were collected and stored at 4 °C and processed within 24 of collection. Primary female HUVECs (FHUVECs) and male HUVEC (MHUVECs) were isolated by collagenase treatment (Sigma-Aldrich, Milano, Italy), as previously described by Addis et al. [25] and cultured in plates pre-coated with 1% gelatine (Sigma-Aldrich, Milano, Italy) in an M199 medium (Life Technologies, Monza, Italy) supplemented with 10% fetal bovine serum (FBS) (Life Technologies, Monza, Italy), 10% newborn calf serum (NBCS) (Life Technologies, Monza, Italy), 1% antibiotic/antimycotic (Sigma-Aldrich, Milano, Italy) and 2 mM of L-glutamine (Sigma-Aldrich, Milano, Italy), until confluence, in a 5% CO2 humidified atmosphere. As previously described (Addis), cultured cells were characterized as endothelial cells by the exhibition of cobblestone morphology when they were contact-inhibited and by an evaluation of the expression of von Willebrand factor, a glycoprotein that is constitutively stored in intra-endothelial Weibel–Palade granules.

FHUVECs and MHUVECs were used in passage 3 to ensure their endothelial characteristics, and all experiments were conducted in duplicate or triplicate.

### 4.4. Cell Viability

FHUVECs and MHUVECs were seeded in a 96-well plate (about 15,000 cells/well in triplicate) and treated with Ang II (0.1–0.5–1 µM) for 24 h. Cell viability was determined by the crystal violet assay according to [88]. The absorbance was recorded at 540 nm and the percentage of viability was calculated in comparison with basal cells, for which a value of vitality equal to 100% has been assumed.

### 4.5. LDH Assay

LDL release was measured in culture medium from basal and cells treated with Ang II (0.1–0.5–1 µM), using the LDH Cytotoxicity Detection kit (Roche Diagnostics, Monza, Italy), following the manufacturer’s instructions. LDL release was expressed as the percentage of the LDH measured in the medium divided by the LDH release measured after cell treatment with 2% Triton X-100 (positive control, 100% LDH release).

### 4.6. Migration Assay 

Migration was evaluated using the chemotaxis assay. HUVECs (6.0 × 10^4^ cells/well) were suspended in M199 medium with or without Ang II (0.5 µM), and placed into the upper chamber of a 24-well modified Boyden Chamber (8.0 µm cell culture inserts; BD Bioscience, Milan, Italy).

Cells inserts with porous membranes were placed over a bottom chamber containing an M199 medium. After 24 h of incubation at 37 °C, the cells that had migrated to the lower side of the filter were stained with DAPI (0.2 mg/mL; Sigma-Aldrich, Milan, Italy), and 5 unit fields per filter were counted using a fluorescence microscope (Motic AE31, Kowloon City, Hong Kong). The percentage of migrated cells was calculated using the ImageJ software (Version 1.53t; NIH, Los Angeles, CA, USA), counting nuclei at each condition in comparison with the baseline. Each sample was examined in duplicate.

### 4.7. MDA Determination

MDA was determined in cell supernatants (100 µL) using the TBARs method as previously described [89] after 24 h of incubation with Ang II (0.5 µM). The quantification was performed spectrophotometrically at 535 nm by measuring the absorbance produced by 100 μL of the sample. Calibration curves were built with standards of MDA at 5, 10, 25, and 50 μM.

### 4.8. Western Blotting

The protein concentration was quantified using the BCA protein assay kit (Thermo Scientific, Waltham, MA, USA). For the Western blot analysis, 25 μg of solubilized protein was electrophoretically resolved by 4–15% SDS-PAGE (100 V, 2 h, 24 °C) and then transferred to a PVDF membrane (250 mA, 65 min, 4 °C) using a Transblot-turbo system (Bio-Rad, Milano, Italy). The membranes were blocked in 5% (*w*/*v*) skim milk (Sigma-Aldrich, Milano, Italy) in 150 mM Tris buffer (Sigma-Aldrich, Milano, Italy) and 20 mM Tris-HCl, pH 7.2 (Sigma-Aldrich, Milano, Italy) at 24 °C for 1 h and then incubated overnight at 4 °C with the following antibodies, all produced in rabbits and diluted at a ratio of 1:1000: α-actin (Sigma-Aldrich, Milano, Italy), LC3 (MBL, Milano, Italy), LAMP1, p62, Beclin-1, and Parkin (Cell Signaling Technology, Milano, Italy). After washing, the blots were incubated for 1 h with horseradish peroxidase (HRP)-conjugated secondary antibody (Cell Signaling Technology, Milano, Italy) (1:2000). Antibody binding was detected using a chemiluminescence reaction (Cell Signalling Technology, Danvers, MA, USA) with the Bio-Rad Chemi Doc instrument (Berkeley, CA, USA). Band volume analysis was performed using the Image Lab 4.0 software (Bio-Rad Laboratories, Berkeley, CA, USA), and densitometric data were normalized on α-actin levels, which did not differ in MHUVECs and FHUVECs [25].

### 4.9. RNA Isolation, Reverse Transcription (RT)-Quantitative (q)PCR Analysis

Total RNA from FHUVECs and MHUVECs were purified using the TriPure isolation reagent (Roche, Merk Life Science, Milano, Italy). First-strand cDNA synthesis was performed using Maxima reverse transcriptase (Thermo Fisher Scientific, Rodano, Italy) and random hexamers and subsequently analyzed by quantitative (q)PCR using SYBR Green mix (Kapa Biosystems, Merk Life Science, Milano, Italy).

The relative mRNA expression levels were calculated by the 2^−ΔCt^ method and *GAPDH* mRNA levels were used for normalization. Gene-specific primer pairs are listed in Table S1.

### 4.10. RNA Isolation, miRNAs Reverse Transcription, and Quantitative (q)PCR Analysis

RNA from FHUVECs and MHUVECs were purified using the TriPure isolation reagent (Roche, Merk Life Science, Milano, Italy) following the manufacturer’s guidelines. miRNA was reverse-transcribed using Mir-X miRNA FirstStrand Synthesis (Takara Bio San Jose, CA, USA) and subsequently analyzed by quantitative (q)PCR analysis using SYBR Green mix (Kapa Biosystems, Merk Life Science, Milano, Italy). Relative levels of miRNAs were calculated by the 2^−ΔΔCt^ method and *U6* RNA levels were used for normalization. Gene-specific primer pairs are listed in Table S2.

### 4.11. Statistical Analysis 

Data were reported as the mean ± standard deviation (SD). A comparison between basal and Angiotensin II-treated cells and between male and female cells in each experimental condition was performed by a t-test using Sigma-Stat 3.1 software (Systat Software, Erkrath, Germany).

The distribution of samples was assessed by Kolmogorov–Smirnov and Shapiro tests. A *p* < 0.05 was considered statistically significant.

## Figures and Tables

**Table 1:** Baseline characteristics of mothers and newborns.

	Male Neonates (*n* = 14)	Female Neonates (*n* = 13)	*p*
Age of mothers (years)	34.5 ± 4.7	35.1± 4.1	NS
Body weight of mothers (start) (kg)	56.6 ± 9.4	56.4 ± 6.4	NS
Body weight of mothers (end) (kg)	66.0 ± 10.2	68.4 ± 7.7	NS
Body mass index (start) (kg/m^2^)	21.4 ± 3.5	21.4 ± 2.1	NS
Body mass index (end) (kg/m^2^)	24.9 ± 4.3	26.0 ± 2.6	NS
Gestational age (weeks)	38.7 ± 1.5	38.7 ± 1.7	NS
Weight of newborns (kg)	3.3 ± 0.5	3.2 ± 0.4	NS

Data are reported as mean ± SD of all subjects enrolled in the study, NS—not significant.

## Data Availability

Data supporting the findings of this study are available from the corresponding author upon reasonable request.

## Supplementary Materials

The following supporting information can be downloaded at: https://www.mdpi.com/article/10.3390/ijms241914929/s1.
