# Supplementary material for: Sex Influence on Autophagy Markers and miRNAs in Basal and Angiotensin II-Treated Human Umbilical Vein Endothelial Cells
Source: Int J Mol Sci. 2023 Oct 5;24(19):14929. doi: 10.3390/ijms241914929 (PMC10573886; doi:10.3390/ijms241914929)
Supplement: Supplementary file 1 [file ijms-24-14929-s001.zip › ijms-2629019-supplementary.pdf]

Supplementary materials

Table S1. Gene-specific primer pairs

RT-(q)PCR primers	Primer Sequences (5′ - 3′)
Park2	FW: CCCTGGGACTAGTGCAGAAT<br>RV: TGCGATCAGGTGCAAAGCTA
Beclin-1	FW:TGAGGGATGGAAGGGTCTAAG<br>RV: GCCTGGGCTGTGGTAAGTAATC
SQSTM1 (p62)	FW: GGGGACTTGGTTGCCTTTT<br>RV: CAGCCATCGCAGATCACATT
LC3	FW: GAGAAGCAGCTTCCTGTTCTGG<br>RV: GTGTCCGTTCACCAACAGGAAG
LAMP-1	FW: CTCTAATGTCTGCAGCTCAAGG<br>RV: TGTACACAGCGCAGAACAGG
GAPDH	FW: TGCACCACCAACTGCTTAGC<br>RV: GGCATGGACTGTGGTCATGAG

Table S2. List of primers used for qPCR

RT-(q)PCR primers	Primer Sequences (5′ - 3′)
miR-29b-3p	TAGCACCATTTGAAATCAGTGTT
miR-126-3p	TCGTACCGTGAGTAATAATGCG
miR-133a-3p	TTTGGTCCCCTTCAACCAGCTG
miR-133b	TTTGGTCCCCTTCAACCAGCTA
miR-223-3p	TGTCAGTTTGTCAAATACCCCA
U6	FW: CTCGCTTCGGCAGCACA<br>RV: CTCGCTTCGGCAGCACA

Table S3. List of references indicating the effect of Ang II on the viability of endothelial cells

Cai S et al Mar Drugs 2021;19(12):655
Han J et al Cell Death Discov 2022;8(1):186
Heng-Jing Hu Mol Med Rep 2016 Nov;14(5):4729-4740
Ijao et al Clin Exp Hypertens 2023; 45(1):2208777
Jian Du Med Sci Monit 2016; 22: 3223–3228
JiaYi Xu Med Sci Monit 2020; 26: e919854-1–e919854-9
Ji C et al Drug Des Devel Ther 2020; 18;14:5087
Li DX et al Gen Physiol Biophys 2020; 39(6):545-555
LI M et al Eur J Pharmacol 2017; 15;797:124-133
Liu B et al Mol Med Rep 2019; 20: 2796-2804
Min L and Liu X Exp Ther Med 2022; 23(2): 132
Song J et al Front Genet 2019; 10: 78.

Wang M et al Ann Clin Lab Sci 2020; 50(3):378-382
Wang Q et al Mol Med Rep 2020; 22(5):4320-4328
Yu Q et al Exp Ther Med 2022; 23(2): 120
Zhang M et al Hum Exp Toxicol 2020; 39(5):734-747

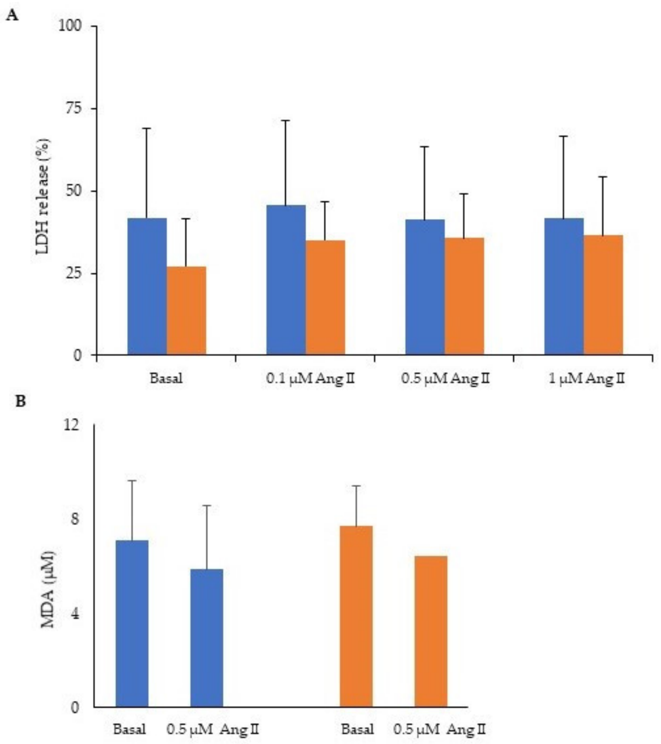

Figure S1. Effect of Ang II on LDH release (A) and MDA levels (B) in MHUVECs (light blue) and FHUVECs (orange) before and after Ang II treatment. Data are reported as mean ± SD of at least 5 subjects per sex
